# Role of thyroid hormone therapy in depressive disorders

**DOI:** 10.1007/s40618-021-01600-w

**Published:** 2021-06-15

**Authors:** M. Bauer, P. C. Whybrow

**Affiliations:** 1 Department of Psychiatry and Psychotherapy, University Hospital Carl Gustav Carus, Technische Universität Dresden, Fetscherstr. 74, 01307 Dresden, Germany; 2 Department of Psychiatry and Biobehavioral Sciences, The Semel Institute for Neuroscience and Human Behavior University of California Los Angeles (UCLA), Los Angeles, CA USA

**Keywords:** Levothyroxine, Triiodothyronine, Thyroid hormone treatment, Bipolar disorder, Major depression

## Abstract

**Purpose:**

The close association among thyroid metabolism, mood disorders and behavior has long been known. The old and modern uses of thyroid hormones to modulate the expression of depression and bipolar disorder and to improve clinical outcome when used in conjunction with psychotropic medications.

**Methods:**

A literature search was performed to identify studies investigating the effects of thyroid hormone treatment in patient s with mood disorders.

**Results:**

The successful modification of mood disorders with thyroid hormone underscores the association between endocrine and cerebral systems in these disorders. Thyroid hormones have a profound influence on behavior and appear to be capable of modulating the phenotypic expression of major mood disorders. In fact, there is evidence that triiodothyronine (LT3) may accelerate the antidepressant response to antidepressants, and studies suggest that LT3 also may augment the response to antidepressants in refractory depression. Add-on treatment with supraphysiologic doses of levothyroxine (LT4) has shown efficacy in open-label and in placebo-controlled studies, including in rapid cycling and prophylaxis-resistant bipolar disorder, and with acute refractory uni- or bipolar depression. Functional brain-imaging studies (PET) demonstrated that administration of supraphysiologic LT4 improves depressive symptoms in patients with bipolar depression by modulating cerebral activity in the anterior limbic network.

**Conclusion:**

The add-on administration of supraphysiologic doses of LT4 is a promising strategy in patients with refractory bipolar and depressive mood disorders.

## Historical background

Interest in using thyroid hormones to treat affective disorders arose from observed associations between psychiatric symptoms and thyroid disease states. Behavioral abnormalities in people suffering from myxoedema were first described in a report from the Clinical Society of London, describing severe mental disturbances including irritability, dementia and severe depression [1]. Nowadays, it is clearly established that in the adult brain disturbances of the thyroid metabolism may profoundly alter mental function, influencing cognition, mood and emotion. In adults, both excess thyroid hormone activity (hyperthyroidism) and inadequate production (hypothyroidism) are associated with changes in intellectual performance, and melancholic depression and dementia are common in severe hypothyroidism [2–4]. The neuropsychiatric impairments accompanying dysfunction of the thyroid axis usually reverse rapidly following return to euthyroid status [5], although there is evidence that severe hypothyroidism, if left untreated, may result in irreversible cognitive disability [6].

Thyroid hormones have been used as treatment since the early twentieth century [7]. With the identification of triiodothyronine (LT3) and levothyroxine (LT4) as the principal thyroid hormones in the 1950s [8], the effect of these hormones, alone and in combination (adjunctive) with standard psychotropic medications, has been studied repeatedly in the treatment of patients with affective disorders, including those without metabolic evidence of peripheral thyroid dysfunction, e.g., hypothyroidism [9].

## Thyroid hormones for treatment of affective disorders

Although it is well established that symptoms of depression that occur in hypothyroid conditions usually resolve after the euthyroid status has been restored by replacement therapy, treatment effects with thyroid hormones alone, without the concomitant use of standard psychiatric medications, have only rarely been studied in affective disorders. The first reports in the late 1950s were case series with inconclusive results [10]. In a later study, Wilson et al. [11] compared LT3 up to 62.5 mcg/d alone with imipramine in depressed patients in a double-blind study. At a dose of 50 mcg/d, LT3 alone was as effective as imipramine. However, later in the study, when LT3 doses reached 62.5 mcg/d, patients showed mild thyrotoxicity, and the study was terminated. Therefore, the study left unanswered the question whether LT3 alone in doses of 50 mcg or less might prove a sufficient treatment for depression. Okuno and Nakayasu [12] administered 50–100 mcg/d of LT4 alone for 2 weeks to patients with primary major depressive disorder without apparent thyroid dysfunction; in aggregate, the average effects were modest, although some patients improved markedly. In summary, there is no systematic research into the question whether thyroid hormones alone are an effective treatment for patients with mood disorder.

## Adjunctive treatment with LT3 and LT4

The use of synthetic thyroid hormones, LT3 and LT4, as adjunctive, supplementary agents in affective disorders, has a long history with the first reports published in the late 1960s/early 1970s [13, 14]. Three groups of studies on the technique of thyroid supplementation of antidepressant or mood-stabilizing drugs must be distinguished: *acceleration* studies, which involve the use of supplementation with thyroid hormone at the initiation of an antidepressant trial to speed up time to response; second, *augmentation* studies, which include the supplementation with thyroid hormone after a 4–8 week trial of an antidepressant has failed or resulted in a partial response. In the third group, the *maintenance* (prophylactic) studies, adjunctive (add-on) treatment with thyroid hormone has been explored to prevent future episodes in recurrent mood disorders.

### Studies with triiodothyronine (LT3)

Prange and associates [13] were the first to investigate the immediate addition of thyroid hormone to speed antidepressant treatment response. To date, seven placebo-controlled studies have been published, five of them showed a positive acceleration outcome. A meta-analysis of double-blind, placebo-controlled studies found that the addition of LT3 had a statistically significant effect on the time to response compared with the effects of placebo [15]. Results of this meta-analysis support an acceleration of antidepressant response when adjunctive LT3 is included early in antidepressant treatment. This analysis also revealed that women may be more likely than men to benefit from the addition of LT3 [15]. Although some of the placebo-controlled studies had some methodological limitations, they were homogeneous with respect to the type of antidepressant (5 of 6 studies used imipramine), the dose of LT3, and outcome measures. Further, studies to assess the efficacy of LT3 acceleration with the newer antidepressants, e.g., selective serotonin reuptake inhibitors (SSRIs), are warranted.

Studies assessing the effects of thyroid hormones in treatment-resistant depression have historically focused largely on LT3 as the augmenting hormone. Case series and at least 13 prospective trials (9 open and 4 controlled double-blind studies) have evaluated the use of LT3 (most studies used 20–50 mcg/d) to potentiate the response to tricyclic antidepressants (TCA) in non-responders to treatment (reviewed in [16]). The open studies consistently showed that about 50% of TCA non-responders are converted to responders within 2–3 weeks after the addition of LT3. However, the controlled double-blind studies are only partially supportive of the data in open studies. As a result, the efficacy of LT3 augmentation remains equivocal. In addition, a meta-analysis did not show consistent results in favor of LT3 augmentation [17]: in aggregating eight prospective studies (either unblinded or double-blinded studies), patients treated with LT3 augmentation were twice as likely to respond as controls. However, improvements in depression scores were not significant among the four double-blind studies. As a result of their meta-analysis, Aronson et al. [17] concluded that additional placebo-controlled data are required for a “definite verdict”. In later placebo-controlled augmentation trials of SSRIs, the results have also been inconsistent: LT3 has been shown to augment the response to sertraline [18] but not to paroxetine [19].

LT3 has not been prospectively studied in the continuation and maintenance treatment of affective disorders. A retrospective chart review (observation period 20 months on average) showed beneficial effects with hypermetabolic doses of LT3 (average dose 90.4 mcg/d) in patients with bipolar disorders [20].

### Studies with levothyroxine (LT4)

Perhaps because of its long half-life (on average 6–7 days), LT4 has not been studied systematically in the *acceleration* of treatment. Thus, compared to the large number of trials on LT3 supplementation, adjunctive treatment with LT4 has been studied less frequently in the treatment of acute depressive disorders. Data from two open trials suggested that augmentation with supraphysiologic doses of LT4 may cause substantial improvement in some patients with treatment-resistant and chronic depression [21, 22]. In an 8-week open study of 17 patients (16 women, 1 man) with treatment-resistant major depressive disorders (bipolar and unipolar), significant improvement was achieved in 50% of patients between week 4 and 8 of treatment with adjunctive LT4 (mean dose 377 mcg/d). Rudas et al. [22] reported in an 8-week augmentation trial with LT4 (mean dose 235 mcg/d) positive effects in six of seven patients with chronic depression/dysthymia. In a single (rater)-blinded 7-week study in 10 euthyroid depressed women with bipolar depression, adjunctive supraphysiologic doses of LT4 (mean dose 320 mcg/day) resulted in a significant improvement of mood (remission in seven patients; partial response in three) [23].

Research from these open-label studies was recently extended in a multicenter, 6-week, double-blind, randomized, placebo-controlled fixed-dose (300 mcg/d LT4) trial to assess efficacy and tolerability of LT4 adjunctive to treatment with a mood stabilizer and/or antidepressant medication for patients with bipolar disorder who were depressed [24]. Patients treated with LT4 did numerically better than those treated with placebo; however, the study failed to detect a statistically significant difference between the two groups in the primary outcome measure (reduction of depression ratings) due to a high placebo response rate in the male participants. Previous findings in the literature that women show better improvement in depression scores with thyroid hormone compared to men were confirmed in a post hoc analysis in the latter study [24].

Furthermore, several open-label studies investigating the prophylactic effects of supraphysiologic LT4 doses in addition to conventional mood stabilizers have suggested that LT4 may improve the course of patients with rapid cycling [25–27] and non-rapid cycling bipolar disorder [28, 29]. In one study that used LT4 alone as a prophylactic medication, Stancer and Persad [25] reported that rapid cycling ceased in 5 out of 8 women with bipolar disorder, but not in two men, following treatment with supraphysiologic doses of LT4 (up to 500 mcg/d). In prospective open studies, investigations demonstrated that *adjunctive* supraphysiologic doses of LT4 may be beneficial in patients with mood disorders resistant to established medications for these disorders [26–29]. For instance, in a 8-year maintenance study, adjunctive treatment of seriously ill and previously prophylaxis-resistant unipolar and bipolar patients with supraphysiologic doses of LT4 proved successful in preventing affective episodes in approximately 60% of the patients: treatment during LT4 treatment compared with the same time period before LT4 administration resulted in a significant reduction of the number of depressive and manic relapses and of the Morbidity Indices [29].

The idea that thyroid hormones may act as modulators in affective illness is further strengthened by studies of the relationship between thyroid function and the clinical course of *bipolar disorder*, especially that of the rapid cycling variant. An intimate association between thyroid homeostasis and bipolar disorder—and particularly rapid cycling—is evident from clinical and research studies [30]. This association prompted the use of thyroid hormone as an adjunct to pharmacologic treatment in those individuals with refractory disease. In an open-label study, the addition of high-dose (supraphysiologic) LT4 to a stable therapeutic regimen of mood-stabilizing drugs in rapid cycling was associated with improvements in depressive symptoms in 10 out of 11 patients and a reduction in manic symptoms in 5 out of 7 patients [26]. Adjunctive treatment with LT4 reduced the manic and the depressive phases in both amplitude and frequency; four patients also underwent single- or double-blind placebo substitution: three patients relapsed after switching to placebo into depression or cycling [26].

Most recently, the first comparative double-blind, placebo-controlled trial of comparing LT4 and LT3 as adjunctive treatments in 32 patients with treatment-resistant, rapid cycling bipolar disorder who had failed a trial of lithium also reported beneficial effects of LT4 [31]. The study provided evidence for the benefit of adjunctive LT4 in alleviating resistant depression, reducing time in mixed states and increasing time euthymic. Adjunctive LT3 did not show statistically significant evidence of benefit over placebo in reducing the time spent in disturbed mood states [31].

## Response to LT4 in the absence of peripheral thyroid disease in affective disorders

Emerging from the studies using supraphysiologic doses of LT4 was the observation that most patients responding to it had no evidence of peripheral thyroid dysfunction. This suggested that in such patients, rather than simply “offsetting” a peripheral thyroid hormone deficit, the high doses of adjuvant LT4 were playing a “central” therapeutic role, directly impacting brain-thyroid homeostasis.

As we have gained more experience with the use of supraphysiologic doses of LT4 in patients with refractory bipolar disease, it has become apparent that many patients who respond to the adjunctive treatment have serum thyroid hormone levels within normal limits, and have no past history of peripheral thyroid disease. Furthermore, there is mounting evidence that these patients respond differently to hypermetabolic doses of LT4 than do healthy control subjects. The doses of LT4 required to achieve the therapeutic effect in these patients are higher (250–600 mcg/d) than those used in the treatment of primary thyroid disorders, e.g., hypothyroidism (typically 75–150 mcg/d) [32]. However, it is well to remember that when LT4 was first introduced into clinical practice in endocrinology in the late 1950s the recommended doses for thyroid hormone replacement in hypothyroidism were far higher (200–400 mcg/d) [33] than those now recommended. But it should be noted that these high doses of LT4 were used before serum TSH assays were available and that such dosing caused significant bone loss [34]. In addition, to be remembered is that supraphysiologic doses of LT4 (up to 1000 mcg/d) or large amounts of desiccated thyroid extract have been administered *for short periods of time* to healthy control subjects without major adverse effects being reported [35].

In the studies described above, patients with refractory affective disorders tolerated high doses of LT4 surprisingly well. To exclude somatic sequelae typically seen in thyrotoxicosis, follow-up studies in LT4-treated patients with affective illness have monitored potentially adverse effects over an extended period of time. One of the concerns with thyroid hormone treatment administered over long periods, as required in prophylaxis, is the increased risk of bone density loss and development of osteoporosis. The potential adverse effects of supraphysiological, TSH suppressive doses of levothyroxine (TSDL) on skeletal integrity are unclear. A review by Biondi and Cooper [36] concludes that there is no evidence that TSDL treatment for differentiated thyroid cancer causes a decrease in bone mineral density (BMD) in males as well as premenopausal women. Findings are controversial for post-menopausal women; calcium, vitamin D and antiresorptive drugs should be considered to reduce the risk of osteoporosis in the latter patient group of patients. Given those facts, we determined whether patients with affective disorders, receiving adjunctive longer term therapy with supraphysiological doses of levothyroxine show evidence of accelerated bone loss compared to the reference population database [37–39]. In summary, our cross-sectional [37] and longitudinal studies [38, 39] in pre- and post-menopausal women and in men did not demonstrate evidence that long-term treatment of affectively ill patients with supraphysiological doses of LT4 significantly accelerates loss of bone mineral density compared to the age-matched reference population. There was no statistically significant difference between the actual percentage decline in bone mineral density and the expected percentage decline in any of the measured bone regions as measured by dual-energy X-ray absorptiometry (DXA) [38, 39]. Addressing cardiovascular tolerability, we systematically monitored changes of cardiac markers prospectively during long-term treatment of mood disorders with supraphysiologic doses of LT4. Since it is well known that hyperthyroidism causes cardiac arrhythmias, e.g., atrial fibrillation, as well as tachycardia, systolic hypertension and especially “high output cardiac failure” we looked for cardiac function based on these markers. In line with cardiologic diagnostic guidelines cardiovascular risk was inferred by repeated comprehensive assessment of echocardiography, cardiac fitness by ergometry and 24-h Holter ECG. During the mean observational period of 20.4 months none of the heart measures reached statistical significance in change over time. None of the assessed cardiac parameters of each single patient was in a range predictive for cardiac dysfunction [40].

In summary, several prospective observational studies have found no evidence for bone loss or increased risk for cardiovascular intolerance during long-term treatment with high doses of LT4. This low incidence of harmful side effects, including bone mineral density and cardiac function, as well as the high tolerability contrasts with the response typically seen in patients with primary thyroid disease who are receiving high-dose thyroid hormone therapy. However, it should be emphasized that in patients not suffering from affective illness, effects of high doses of LT4 on the skeleton [41, 42] and heart [43] is a real phenomenon, although the effect on bone density is more variable [44, 45].

## Putative central hypothyroidism: causal mechanism of good tolerability?

As part of an ongoing effort to define the effects on other behavioral domains [46], tolerability and safety parameters of supraphysiologic doses of LT4, we have also observed under well-controlled conditions significant differences in the physiological response of healthy control subjects and depressed patients [47]. The peripheral thyroid hormone indices (total T_4_, free T_4_; total T_3_, free T_3_) in refractory depressed patients were less elevated in response to high doses of LT4 than is the case in healthy controls and the patients suffered significantly fewer side effects [48], suggesting a syndrome of resistance to thyroid hormone. Thus, the possibility arises that while a subgroup of patients with affective disorder develop refractory illness and resistance to standard psychotropic agents because of diminished CNS thyroid hormone availability secondary to peripheral thyroid disease (which is sometimes exacerbated by lithium carbonate treatment), others have a diminished central (i.e., brain) capacity to *utilize* thyroid hormones. In both instances, the use of LT4 in supraphysiologic doses increases the availability of thyroxin substrate to the brain and improves the CNS thyroid economy: in the case of those with peripheral thyroid dysfunction by restoring a euthyroid state, and in those where no peripheral disturbance is evident by overriding the putative central resistance to thyroid hormone. The regulation of thyroid hormone homeostasis in the brain underlies a complex interaction of different mechanisms, including the activity of specific thyroid hormone transporters (e.g., monocarboxylate transporter 8, MCT8) and the carrier transthyretin that are involved in determining intracellular concentrations of thyroid hormones. Deiodinases control regional effectivity of T3 in concert with other mechanisms, i.e., the local distribution of the different nuclear thyroid hormone receptors TRα and TRβ which are widely distributed in the brain with high concentrations in cerebral cortex, hippocampus and amygdala [49], the latter being limbic structures implicated in the pathogenesis of affective disorders. Deficits in any one, or several, of these mechanisms may result in reduced bioavailability of thyroid hormones at cerebral target regions despite normal peripheral serum levels of thyroid hormones. This condition has been conceptualized as “central hypothyroidism”: compensation for this condition might be one reason why a proportion of euthyroid depressed patients benefit from administration of supraphysiologic doses of LT4 and tolerate it well without serious adverse effects [23, 50].

## Thyroid hormones and brain metabolism in functional imaging studies

So what evidence is there to support this conjecture of a central disturbance of brain–thyroid metabolism in (some) patients who suffer refractory affective illness? Following the lead of the above-described clinical studies, we investigated the effects of adjunctive supraphysiologic doses of LT4 on regional relative brain activity using as a surrogate index of cerebral glucose metabolism measured by positron emission tomography (PET) with [F-18]fluorodeoxyglucose (FDG) in (euthyroid) women with refractory bipolar depression. At baseline (pretreatment), bipolar depressed women had functional abnormalities in prefrontal and limbic brain areas compared to healthy controls. Over 7 weeks, the treatment with LT4 significantly improved mood and was accompanied by significant changes in relative brain activity. In particular, LT4 treatment was associated with a widespread relative deactivation of limbic and subcortical structures, including the amygdala, hippocampus, caudate nucleus, ventral striatum, thalamus and cerebellar vermis [23]. We extended this work in a randomized, double-blind, placebo-controlled study of patients with bipolar depression in which cerebral glucose metabolism was again assessed with FDG-PET before and after 6 weeks of treatment with LT4 [50]. In these studies, adjunctive LT4 treatment produced a significant decline in depression scores during the 6-week treatment. Furthermore, in patients treated with LT4, we found a significant decrease in regional activity in the left thalamus, right amygdala, right hippocampus, left ventral striatum and right dorsal striatum. Decreases in the left thalamus, left dorsal striatum and subgenual cingulate were correlated with a reduction in depression scores. The two treatment groups (LT4 and placebo) differed significantly in the relationship between the changes in depression scores and in activity in the thalamus bilaterally and the left ventral striatum [50].

Thus, the findings provided evidence that administration of supraphysiologic LT4 improves depressive symptoms in patients with bipolar disorder by modulating function in components of the anterior limbic network components that have been implicated in the pathophysiology of mood disorders. These studies, which are the first to use PET technology to demonstrate the effects of treatment with LT4 on regional brain metabolism in patients with bipolar disorder, confirm that thyroid hormones are capable of modulating metabolic function in the mature adult brain, and provide some intriguing neuroanatomical clues as to the locus of that action.

## Searching for a brain–thyroid metabolic deficit in affective disorders

The investigation of a potential thyroid–brain metabolic deficit in those patients with treatment-resistant affective disorder is a focus of future research in this area. The key steps of cerebral thyroid metabolism should be considered: the uptake of thyroid hormones into brain; the production of the active hormone LT3; thyroid receptor activity; and the genetics that underpin these various functions [49]. Interactions of thyroid hormones and the neurotransmitter systems of the brain mainly norepinephrine and serotonin, which play a major role in the modulation of mood and behavior, are also important although the specific mechanisms through which their influence occurs are unclear [51, 52]. However, there is robust evidence, particularly from animal studies, that the thyroid status has a modulating impact on the serotonin system in the developing and mature brain. Thus, exogenously administered thyroid hormones may exert their modulatory effects in affective illness via fostering an increase in serotonergic neurotransmission, specifically by reducing the sensitivity of 5-HT_1A_ autoreceptors in the raphe nuclei, and by increasing 5-HT_2_ receptor sensitivity [52]. Whether these 5-HT receptor modulations represent the final common pathway for behavioral change in the affective illness remains to be elucidated, but it is a potentially fruitful line of future research.
